# Glycomics using mass spectrometry

**DOI:** 10.1007/s10719-012-9376-3

**Published:** 2012-04-25

**Authors:** Manfred Wuhrer

**Affiliations:** Department of Parasitology, Biomolecular Mass Spectrometry Unit, Leiden University Medical Center, Albinusdreef 2, 2333ZA Leiden, The Netherlands

**Keywords:** Cancer, Congentical disorders of glycosylation, Lysosomal storage diseases, MALDI-TOF-MS, Permethylation

## Abstract

Mass spectrometry plays an increasingly important role in structural glycomics. This review provides an overview on currently used mass spectrometric approaches such as the characterization of glycans, the analysis of glycopeptides obtained by proteolytic cleavage of proteins and the analysis of glycosphingolipids. The given examples are demonstrating the application of mass spectrometry to study glycosylation changes associated with congenital disorders of glycosylation, lysosomal storage diseases, autoimmune diseases and cancer.

## Introduction

Mass spectrometry (MS) based glycomics techniques are broadly used to analyze free oliogsaccharides, glycosaminoglycans as well as the glycan portions of glycoproteins, proteoglycans and glycolipids. A wide range of MS equipments are available for glycoconjugate analysis. Both matrix-assisted laser desorption-ionization (MALDI) and electrospray ionization (ESI) are commonly applied. MS may be used as a stand-alone technique, or coupled online to separation methods such as HPLC [1–4] and capillary electrophoresis (CE) [5–7]. Carbohydrate and glycoconjugate analysis by MALDI-MS has been comprehensively reviewed by Harvey [8, 9]. Other useful review articles, which cover a range of analytical techniques including tandem MS (MS/MS) of glycoconjugates have appeared in recent years [8–15].

This review aims at giving a concise overview of MS based glycomics technology, together with selected applications in clinical research.

## Analysis of free glycans

Protein-linked N-glycans and O-glycans are typically released by enzymatic and chemical methods, respectively [16]. Also glycosaminoglycans are generally degraded by chemical or enzymatic means for subsequent analysis [5, 17]. Analysis of released (or “free”) glycans may be achieved by a variety of techniques such as mass spectrometry, HPLC of reductively aminated glycans employing fluorescence or UV detection and capillary gel electrophoresis with laser-induced fluorescence detection (CGE-LIF) of labeled glycans [16, 18–21]. MS is particularly advantageous for analyzing very complex glycan mixtures containing unusual oligosaccharide structures for which the standardized migration positions in HPLC or CGE-LIF have not yet been determined. Importantly, the mass of the analyzed glycan – when determined with sufficient accuracy or accompanied by a tandem MS experiment – will directly provide information on the glycan composition in terms of hexoses, *N*-acetylhexosamines, deoxyhexoses, etc. By contrast, this direct link between the observed glycan species and its molecular composition is not inherently present for HPLC and CGE-LIF experiments and additional efforts are required such as the use of glycan standards or exoglycosidase treatments for the determination of terminal monosaccharides [22, 23]. On the other hand, separation-based methods for glycan analysis will often resolve structural isomers such as the 6-arm and 3-arm isomers of monogalactosylated biantennary glycans [24], while their distinction is not easily achieved by MS and requires additional efforts such as tandem MS analysis [25].

Therefore, while very complex pools of oligosaccharides can be analyzed by MS(/MS) without separation [26], many researchers choose to perform glycan analysis by LC-MS [1–3] or - less frequently - CE-MS coupling [5–7]. (Normalized) retention and migration times, precursor masses and fragmentation spectra may then be used for structural elucidation as in the case of O-glycan alditol analysis by porous graphitized carbon (PGC) HPLC coupled online to MS [27–29]. PGC-HPLC appears to have a particularly high power in separating oligosaccharide structural isomers, which makes this method very useful for in-depth structural analysis of complex oligosaccharide mixtures [2]. Another popular separation technique hyphenated with MS for oligosaccharide analysis is HILIC, which likewise features isomer separation [24, 30, 31]. High-performance anion-exchange chromatography (HPAEC) coupled with online-desalting and online-ESI-MS is another approach which is particularly useful for the analysis of underivatized oligosaccharides [32].

Derivatization is often useful to support mass spectrometric detection and identification of carbohydrates [33]. For example, oligosaccharides may be reduced to alditols resulting in a 2 Da mass tag on the innermost monosaccharide which facilitates fragment assignment in tandem MS. Analysis of O-glycan alditols obtained by reductive beta-elimination may be achieved by porous graphitized carbon (PGC) HPLC coupled via online, negative-mode electrospray ionization to ion trap-tandem mass spectrometry (MS/MS) [27, 28]. An online database has been made available by the UniCarb-DB partners allowing structural assignment of O-glycan alditols on the basis of MS and MS/MS spectra in addition to retention times (http://www.unicarb-db.com/). Similarly, N-glycans may be structurally assigned on the basis of mass and retention time in PGC-ESI-MS. This approach has been introduced by Altmann and coworkers [29].

Within the range of mass spectrometric techniques, negative-mode MS of glycans has recently obtained increased attention, both for MALDI and ESI ionization [33, 34]. There are several attractive features of analyzing glycans in negative-ion mode. Negative-mode ionization is particularly effective for acidic glycan structures. In this respect, labeling of glycans at the reducing end with an acidic tag such as 2-aminobenzoic acid (anthranilic acid; AA) is advantageous, as it confers acidic properties to all glycans including neutral species, thereby allowing the efficient detection of both sialylated and non-sialylated AA-labeled oligosaccharides in negative-mode MALDI-time of flight (TOF)-MS [16]. In addition, negative-mode MS/MS of oligosaccharides has attractive features, for example that the glycosidic linkages of fucose are rather stable, in contrast to their labile behavior in positive-ion mode [35]. Harvey has described several diagnostic ions, which are observed in negative-ion mode MS/MS of N-glycans and allow the elucidation of antenna compositions as well as the differentiation between the 6-branch and 3-branch of the glycan [36, 37].

**Fig. 1:**
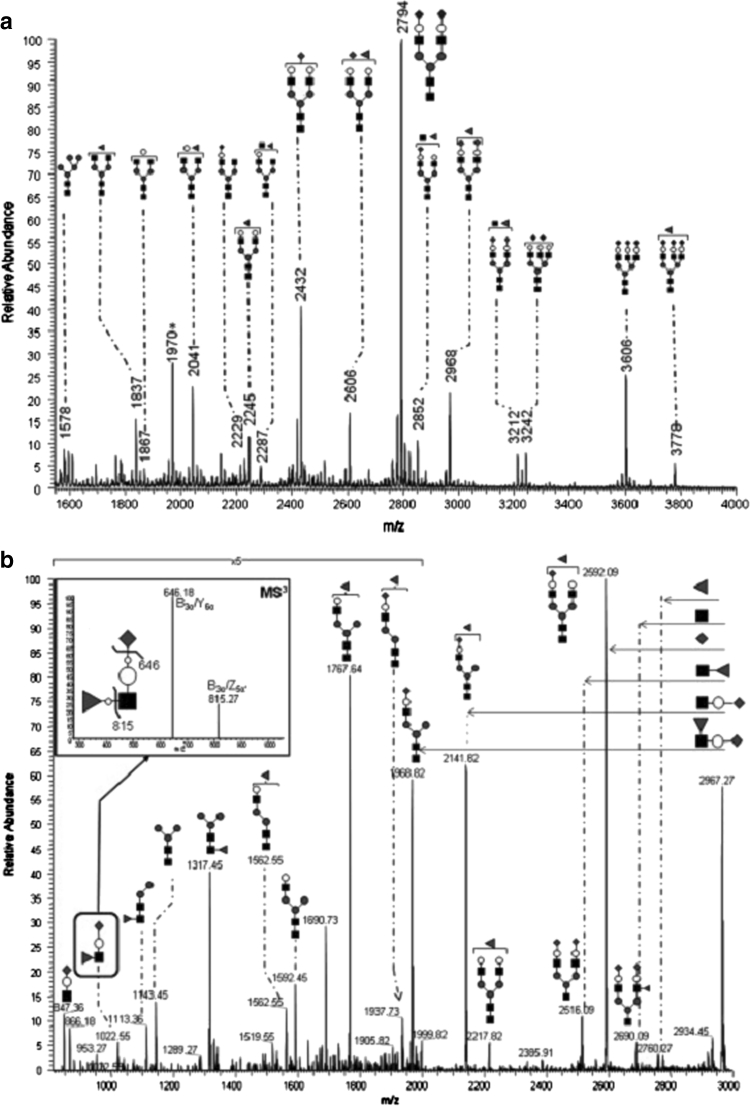
Permethylated serum N-glycans measured by MALDI-linear ion trap-MS (**a**). Tandem mass spectrum of the sodiated precursor ion at m/z 2968. Fragments at *m/z* 1022, 1143, and 2690 are indicative of antenna fucosylation, whereas fragments at *m/z* 1113, 1317, and 2516 mark core fucosylation (**b**). The inset in (**b**) shows an MS3 experiment of m/z 1022 confirming the proposed structurewith terminal sialic acid and antenna fucosylation. Filled square, GlcNAc, empty circle, galactose; filled circle, mannose; filled triangle, fucose; filled diamond, N-acetylneuraminic acid. Taken from [39] with permission

Alternatively, oligosaccharides may be analyzed after permethylation [33]. Permethylation converts all the hydroxyl groups into methyl ethers. Moreover, the carboxylic acid groups of sialylated glycans are protected by methyl esterification, which stabilizes the sialic acids and enables MALDI-TOF-MS profiling of permethylated neutral and acidic glycans simultaneously. By contrast, sialic acids are labile when analyzing native glycans, leading to the observation of degradation products in MALDI-TOF-MS spectra [16, 33]. Analysis of the sodium adducts of permethylated glycans by tandem MS is a very useful approach for detailed structural characterization as - next to cleavages of glycosidic bonds - diagnostic cross-ring cleavages are observed, which reveal linkage positions. These analyses may be performed by high-energy collision-induced dissociation (CID) MALDI-TOF/TOF-MS resulting in very complex yet informative fragmentation spectra [38]. MALDI-ion trap-MS of permethylated N-glycans released from total plasma glycoproteins has recently been established by Guillard *et al.* [39]. This approach allows in-depth analysis of glycans by multistage-tandem mass spectrometry as exemplified in Fig. 1: MS2 (Fig. 1a) and MS3 experiments (Fig. 1b) provided evidence for the occurrence of a sialyl-Lewis X structure on a plasma N-glycan.

Analysis of permethylated glycans in combination with ESI-ion trap-MS is particularly attractive. When this approach is combined with multistage fragmentation of permethylated glycans, the combination of various characteristic fragmentation spectra of sub-structures of the precursor oligosaccharides allows the unambiguous structural assignment of large oligosaccharide structures as impressively demonstrated by Reinhold and coworkers [25, 26].

Internal standards for MS may be obtained by isotope labeling during the derivatization step. For example reductive amination or permethylation using deuterated or C13-labeled versions of the tag / chemicals have been shown to be advantageous for oligosaccharide quantification and the detailed comparison of glycan profiles [16]. It has to be noted, however, that most of these isotope labeling strategies have not yet been applied to clinical glycomics research questions.

## Analysis of glycopeptides

In addition to the analysis of released glycans studying protein glycosylation at the level of glycopeptides is rapidly gaining importance [40–44]. The peptide portion may be seen as a tag, which potentially allows the assignment of the glycan to a specific N- or O-glycosylation site on a specific protein. However, this approach is complicated by several obstacles. First, proteolytic cleavage is often hindered in highly glycosylated proteins, resulting in very large, highly and heterogeneously glycosylated peptide moieties, which are hardly accessible for MS analysis [45]. Second, a variety of glycans are generally found attached to one specific glycosylation site (microheterogeneity of glycosylation), and different N-glycosylation sites on one protein often have different glycan patterns. Therefore, glycopeptides generally occur substoichiometrically, making them difficult to analyze by MS in the presence of a majority of non-glycosylated peptides. Various enrichment techniques including lectin affinity chromatography are available to purify glycopeptides for MS analysis [3, 46]. A very promising technique for enriching N-glycopeptides is hydrophilic interaction liquid chromatography-solid phase extraction (HILIC-SPE), which may be performed using silica-based or carbohydrate-based stationary phases [30, 31, 47, 48]. Third, depending on the size of the glycan moiety and the chosen MS/MS approach, it is often hard to obtain peptide sequence information, which is in most cases needed for unambiguous assignment of the glycan to a specific protein [46]. Popular approaches are electron capture dissociation (ECD) and electron transfer dissociation (ETD) of glycopeptides as well as various types of (multistage) CID [4, 43, 44]. In ECD and ETD the glycan portion is generally stable, and peptide backbone cleavages tend to provide (some) peptide sequence information [4]. Single stage low-energy CID (as occurring on an ion trap) is generally characterized by fragmentation of glycosidic bonds, and peptide backbone cleavages are usually minor, if detectable at all. Fragmentation of the peptide portion may be achieved by performing ion trap-multistage MS/MS, and has been successfully applied in various cases for the identification of glycosylated proteins and glycosylation sites [41, 43]. Alternatively, fragmentation of glycopeptides at elevated energies in MALDI-TOF/TOF-MS and MALDI- or ESI-quadrupole-TOF-MS has been reported to provide peptide sequence information next to information on glycan composition and structure [4].

Glycopeptide analysis is almost exclusively performed on protonated species in positive-ion mode. It has been observed that under these conditions glycan moieties may undergo rearrangements in MS/MS, of which prominent examples are the migration of fucoses between N-glycan antennae, or from the core to outer portions of the N-glycan structure [49, 50]. These rearrangements may not only be observed for N-glycopeptides, but also for O-glycopeptides. Obviously, awareness of these processes is required for avoiding misinterpretation of glycopeptide fragmentation spectra.

The major bottle-neck in glycopeptidomics-based proteomics of complex samples is data analysis. Software supporting data analysis is desperately needed, and several promising approaches have recently been reported ([45] and references cited therein). Yet additional, concerted efforts in developing data analysis tools are needed to boost the impact of this analytical approach.

Analyzing protein glycosylation at the glycopeptide level may be categorized as part of a bottom-up glycoproteomics approach. The analysis of the intact mass of glycoproteins, together with a bottom-up analysis, often allows the detailed structural assignment of protein species such as monoclonal antibodies [51, 52]. In addition, top-down glycoproteomics, *i.e.*, the MS analysis of intact glycoproteins followed by their tandem MS analysis for the characterization of posttranslational modifications including glycosylation, has high potential but needs to be further developed [53, 54].

**Fig. 2:**
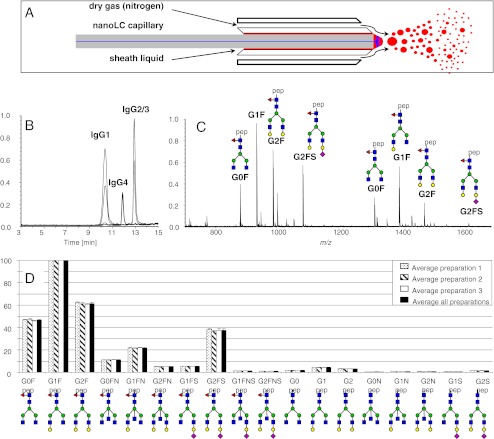
IgG Fc-glycosylation profiling by LC-quadrupole-TOF-MS. Long-term stability of MS detection in nanoLC-MS is achieved using a sheath-flow ESI spray with a sheath flow of 2 μl/min 50 % isopropanol, 20 % propionic acid (**a**). The extracted ion chromatograms of IgG1, IgG4 and IgG2/3 tryptic Fc glycopeptide species (**b**), and the mass spectrum of the IgG1 Fc glycopeptides elution range is shown in (**c**). Glycopeptide signals observed below *m/z* 1200 are triple protonated, and signals above *m/z* 1200 are double protonated. The excellent repeatability of the overall sample preparation and analysis method is demonstrated in (**d**) for IgG1. *Blue square*, *N-*acetylglucosamine; *red triangle*, fucose; *green circle*, mannose; *yellow circle*, galactose; *purple diamond*, *N-*acetylneuraminic acid

Glycopeptide analysis by MS can be performed in a high-throughput mode. IgG glycopeptide profiling by MALDI-TOF-MS has been performed to determine the changes in IgG1 and IgG2 Fc glycosylation features with pregnancy and rheumatoid arthritis [55] as well as with longevity and healthy aging [56]. MALDI-FTICR-MS was likewise evaluated for IgG Fc glycopeptides profiling and was found to be particularly useful for analyzing changes in sialylation [57]. MALDI-FTICR-MS analysis of IgG Fc glycopeptides is characterized by reduced losses of sialic acid, which is most probably due to the higher pressure in the source and the resulting collisional cooling, in combination with the lower extraction voltages as compared to MALDI-TOF-MS [57]. Recently, using a sheath-flow ESI sprayer, a robust nanoLC-MS method for IgG Fc glycosylation profiling was established [58] (Fig. 2). Notably, the sheath-flow ESI sprayer setup was found to significantly increase the long-term stability of the system while keeping the sensitivity of the system in the same range as with conventional nano-ESI-MS [58]. High-sensitivity IgG Fc glycosylation analysis is particularly valuable when analyzing affinity-purified, antigen-specific IgGs, which may be present at low concentrations. For the most common applications, however, such as glycosylation analysis of total plasma IgG and biotechnologically produced IgG the available sample amounts are generally plenty and sensitivity is not an issue. The sheath flow setup was used in combination with trifluoracetic acid containing running solvents resulting in the coelution of sialylated and non-sialylated IgG Fc glycopeptides. In contrast, conventional nano-LC-MS with formic acid-containing running solvents features early-eluting glycopeptides with neutral glycans and late-eluting ones with sialylated glycans [58]. This set-up was used to study IgG Fc glycosylation changes during pregnancy. It was found that galactosylation, sialylation were increased whilst fucosylation and the incidence of bisecting GlcNAc were decreased during pregnancy. The observed glycosylation changes may contribute to the immune suppression occurring during pregnancy in order to protect the fetus from alloimmune reactions of the mother [58].

## Analysis of glycolipids

Next to glycoproteins, glycolipids play an important role in cellular interaction and cellular differentiation [59]. The majority of glycolipids observed in humans have a ceramide portion (sphingoid base carrying an amide-linked fatty acid) and are, therefore, categorized as glycosphingolipids. They occur mainly in the outer leaflet of the plasma membrane and also in the inner membranes. Glycosphingolipids show a marked tissue- and cell type-specific expression pattern [59, 60]. This is for example reflected by the fact that various human cluster of differentiation (CD) markers, which are differentially expressed between leukocytes, are glycolipids such as CD60a (GD3; Neu5Ac(α2-8)Neu5Ac(α2-3)Gal(β1-4)Glc(β1-1)ceramide), CD60b (9-*O*-acetyl GD3), CD60c (7-*O*-acetyl GD3), CD77 (Gb3; globotriaosylceramide; Gal(α1-4)Gal(β1-4)Glc(β1-1)ceramide)) (http://www.hcdm.org/).

Glycosphingolipids are generally subjected to MS in intact form [61]. Recently, chip-based approaches for glycosphingolipid analysis have been reviewed [59, 62]. Importantly, there is also technology available to combine the most commonly applied separation technique in lipid, as well as glycolipid analysis, high-performance thin layer chromatography (HPTLC), with MS. For example, glycosphingolipids separated by HPTLC can be probed by overlay detection using carbohydrate-binding proteins such as lectins, bacterial toxins, and antibodies, followed by the MS analysis of positive bands, either directly from the HPTLC plate or after lipid extraction, as reviewed recently by Meisen *et al.* [63]. Alternatively, glycolipids may be analyzed by HILIC-nano-LC-MS [64] using slightly adjusted solvent conditions when compared to methods used for glycan and glycopeptide separation [30]. Using this approach, it has been demonstrated that α2-3-sialylated and α2-6-sialylated isomers of lactoneotetraosylceramides can be baseline separated from complex mixtures and characterized individually by tandem MS [64].

## Clinical glycomics applications

The importance of the above described techniques is illustrated by their application in clinical studies. Glycosylation changes play important roles in the cellular mechanisms of health and disease [65], and glycans have a great potential as biomarkers for different types of cancer [66, 67]. There is a vast range of studies of human glycobiology in healthy and diseased people employing MS, and some selected examples will be presented demonstrating the potential of mass spectrometric approaches for clinical glycomics.

MS has been shown to be useful to type congenital disorders of glycosylation. Guillard *et al.* established an approach that relies on N-glycan release from total plasma, permethylation, and MALDI-ion trap-MS measurement [39], allowing in-depth analysis of glycans by tandem MS (Fig. 1). This approach was applied to determine plasma N-glycan profiles of congenital disorder of glycosylation (CDG) type II patients, as well as controls [68]. A total of 38 peaks were assigned in terms of molecular composition, and changes in the N-glycan profiles were found to be useful to distinguish between the patient groups. The authors also successfully addressed the challenge of differentiating CDG type II diseases from other diseases with secondary causes of underglycosylation. This method is now being successfully applied in clinical research, including research on patients with defects in 1-4-galactosyltransferase I (B4GAT1), which leads to the expression of largely truncated glycans on plasma proteins [69].

**Fig. 3:**
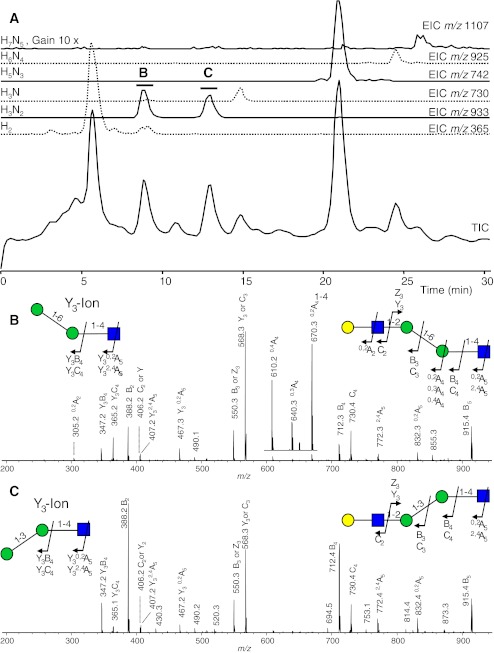
High performance-anion exchange chromatography with online MS detection of urinary oligosaccharides of a GM1-gangliosidosis patient (**a**). Next to the total ion chromatogram (TIC) specific extracted ion chromatograms are given labeled with the composition of the oligosaccharide in terms of hexoses (H) and N-acetylhexosamines (N). The ion trap tandem mass spectra obtained for the two detected H3N2 isomers are shown in (**b**) and (**c**). *Green circle*, mannose; *yellow circle*, galactose; *blue square*, N-acetylglucosamine. Fragment ions are assigned according to Domon and Costello [74]. Taken from [32] with permission

Another application field for MS is represented by the analysis of lysosomal storage disorders. Lysosomal defects of glycoconjugate degradation may lead to the secretion of glycopeptides, glycolipids or oligosaccharides in patient urine. These secreted molecules are potential markers of the diseases. Molecular analysis of these degradation products by MS often directly pinpoints to the genetic defect. In Schindler’s disease, which is a hereditary *N*-acetylhexosaminidase deficiency, characteristic O-glycosylated amino acids and O-linked glycopeptides were detected from patients’ urines [70]. In Fabry’s disease, the causative enzymatic defect leads to elevated levels of globotriaosylceramide and lyso-globotriaosylceramide species in urine and plasma, which can be detected by LC-MS with good diagnostic sensitivity and specificity [71, 72]. A very powerful approach for the analysis of urinary oligosaccharides is HPAEC, which was applied in capillary-scale with online-desalting and ESI-ion trap-MS/MS analysis to study urinary oligosaccharides of patients with GM1-gangliosidosis and galactosialidosis [32, 73]. On the basis of literature knowledge of N-glycan biosynthesis, this approach allowed the structural assignment of chromatographically separated isomeric N-glycan degradation products in GM1-gangliosidosis (Fig. 3). The observation of N-glycans with terminal galactose residues points to a deficiency of β-galactosidase activity [32]. When the same analytical setup was applied to study urinary glycans in galactosialidosis, novel degradation products were observed such as glycolipid-derived oligosaccharides, both in reducing form and with C1-oxidation of the innermost glucose [73]. These results indicate the presence of an alternative glycolipid degradation pathway in galactosialidosis patients involving a hitherto not described endoglycoceramidase activity.

The analysis of protein degradation products from bio fluids has repeatedly led to the identification of glycopeptides, thereby shedding new light on protein glycosylation. For example, apolipoprotein CIII-derived O-glycopeptides were found in the urine of *Schistosoma mansoni* infected individuals [75]. Remarkably, these glycopeptides did not exhibit the sialylated T-antigen glycan structures found on apolipoprotein CIII from human serum, but instead carried larger O-glycan structures with a high degree of sialylation. In another study, an O-glycosylated peptide stemming from the C-terminus of the fibrinogen α-chain was found to be increased in the urine during urinary tract infection with *Escherichia coli* [76]. Recently, O-glycosylated amyloid β-peptides representing a potential disease biomarker were characterized from cerebrospinal fluid of Alzheimer patients using both CID and ECD fragmentation [41].

Cancer glycomics biomarker discovery has recently been reviewed [66, 67], and MS is becoming an important research tool in this field. Novotny and Mechref with coworkers chose to analyze serum N-glycan profiles after permethylation using MALDI-TOF-MS. Using this approach, they demonstrated vastly different N-glycan profiles in metastatic prostate cancer as compared to healthy tissue [77]. A variety of mainly fucosylated, complex-type N-glycans were found to be increased in cancer *vs*. control. In another study the relative abundances of a set of 8 complex-type serum N-glycans were found to be indicative of the progression of breast cancer [78]. Other studies have focused on the glycosylation analysis of specific acute-phase proteins. For example, MALDI-MS of 2-aminobenzoic acid-labeled N-glycans showed that the N-glycan fucosylation of α-1-acid glycoprotein is significantly increased in ovarian cancer [79]. Notably, most of the reported cancer glycomics studies focus on the analysis of the total plasma or serum N-glycome or certain acute-phase proteins [66, 67]. While these approaches are promising, an increase in sensitivity and specificity may be expected when tumor-derived antigens isolated from body fluids are characterized together with their specific glycosylation profiles.

**Fig. 4:**
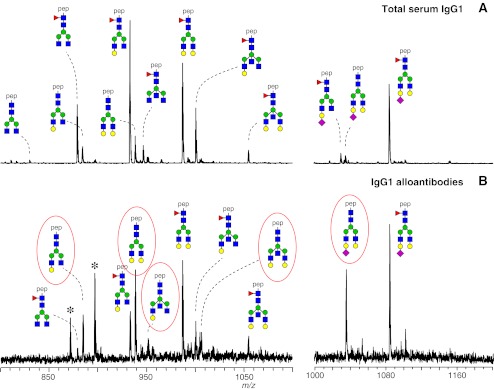
Low core-fucosylation of anti-HPA-3a alloantibodies. Fc glycosylation of total serum IgG1 (**a**) and anti-HPA3a alloantibodies from a patient with pregnancy complications (fetal and neonatal alloimmune thrombocytopenia; FNAIT) (**b**). Glycopeptides were detected in triple protonated form by nanoLC-ESI-ion trap-MS carrying neutral N-glycan chains (left panels) and acidic N-glycan chains (right panels). In (**b**) the assigned structures representing afucosylated glycoforms are highlighted in *red*. *Blue square*, N-acetylglucosamine; *yellow circle*, galactose, *green circle*, mannose; *red triangle*, fucose; *purple diamond*, N-acetylneuraminic acid; pep, tryptic peptide moiety; asterisk, non-glycopeptide signal. Taken from [82] with permission

Still another glycomics application area for MS is the study of the genetic and environmental regulation and dysregulation of protein glycosylation in health and diseases [80]. For example, various novel aspects of the regulation of immunoglobulin G Fc glycosylation have only recently been revealed by high-sensitivity glycosylation profiling at the glycopeptide level. Employing this analysis technique, *in vitro* studies have shown that soluble factors such as cytokines and toll-like receptor ligands modulate the degree of IgG Fc galactosylation, sialylation and the incidence of bisecting GlcNAc [81]. Likewise, fucosylation of IgG Fc glycans appears to be regulated in humans: IgG Fc glycan fucosylation in humans is known to be generally above 90 %, yet recently pathogenic alloantibodies with a low degree of fucosylation (50 % and below) have been described for patients with fetal and neonatal alloimmune thrombocytopenia (FNAIT) [82]. Figure 4 shows the total serum IgG1 Fc glycosylation profile of a patient and the corresponding profile of the pathogenic anti-human plate antigen (HPA) 3a alloantibodies. While the total serum IgG1 shows 9 % afucosylated structures (A), the afucosylation is 38 % for the alloantibodies of this patient (B). Importantly, these IgG Fc glycosylation changes are known to be functionally relevant. Low fucosylation has been associated with enhanced cellular cytocoxicity [83], whilst high degrees of sialylation confer anti-inflammatory properties to IgGs [84].

## Perspectives

In the coming years the field of mass spectrometric analysis of protein glycosylation is expected to show an increase in measurement sensitivity and precision as well as sample throughput, allowing the in-depth analysis of biological systems, with data analysis being a major challenge [44].

Despite the limitations mentioned in this review, glycoproteomics approaches focusing on the glycopeptide level will gain in popularity. Mass spectrometric analyses of (tryptic) glycopeptides are rewarding as they have intrinsically the potential of assigning specific glycan structures to a specific site on a specific protein. This information is often of utmost importance, as the primary role of glycans is modulating the properties (such as function, activity, stability, targeting) of their carrier proteins. Notably, approaches based on the analysis of released N-glycans often fail to provide this information on protein- and site-specificity and are, therefore, of limited value.

Another important aspect is the analysis of intact glycoproteins by MS, which is expected to gain importance in the next years. On the one hand, MS analysis of intact glycoproteins allows the integration of the information obtained at the glycopeptide and released glycan level to obtain an overall view of protein glycosylation [51]. On the other hand intact protein analysis may be accompanied by top-down tandem mass spectrometric analysis for characterization of posttranslational modifications, including glycosylation [85].

It is anticipated that the concept of a specific protein having specific functions will undergo refinement, and specific proteins will be perceived as an assembly of isoforms (including glycoforms) that are caused by a variety of posttranslational modifications including glycosylation. Defining such “protein species” is of utmost importance for functional proteomics supporting systems biology [86] and will require bioinformatics tools and databases to facilitate posttranslational modification analysis at the glycopeptide level [44].

## References

[1] Pabst M, Altmann F (2011). Glycan analysis by modern instrumental methods. Proteomics.

[2] Ruhaak LR, Deelder AM, Wuhrer M (2009). Oligosaccharide analysis by graphitized carbon liquid chromatography-mass spectrometry. Anal. Bioanal. Chem..

[3] Wuhrer M, Deelder AM, Hokke CH (2005). Protein glycosylation analysis by liquid chromatography-mass spectrometry. J. Chromatogr. B.

[4] Wuhrer M, Catalina MI, Deelder AM, Hokke CH (2007). Glycoproteomics based on tandem mass spectrometry of glycopeptides. J. Chromatogr. B Analyt. Technol. Biomed. Life Sci..

[5] Zaia J (2009). On-line separations combined with MS for analysis of glycosaminoglycans. Mass Spectrom. Rev..

[6] Mechref Y (2011). Analysis of glycans derived from glycoconjugates by capillary electrophoresis-mass spectrometry. Electrophoresis.

[7] Mechref Y, Novotny MV (2009). Glycomic analysis by capillary electrophoresis-mass spectrometry. Mass Spectrom. Rev..

[8] Harvey, D.J.: Analysis of carbohydrates and glycoconjugates by matrix-assisted laser desorption/ionization mass spectrometry: an update for 2007–2008. Mass Spectrom. Rev. **31,** 183–311 (2012) 10.1002/mas.20333 21850673

[9] Harvey DJ (2011). Analysis of carbohydrates and glycoconjugates by matrix-assisted laser desorption/ionization mass spectrometry: an update for the period 2005–2006. Mass Spectrom. Rev..

[10] Zaia J (2008). Mass spectrometry and the emerging field of glycomics. Chem. Biol..

[11] North SJ, Hitchen PG, Haslam SM, Dell A (2009). Mass spectrometry in the analysis of N-linked and O-linked glycans. Curr. Opin. Struct. Biol..

[12] Morelle W, Canis K, Chirat F, Faid V, Michalski JC (2006). The use of mass spectrometry for the proteomic analysis of glycosylation. Proteomics.

[13] Morelle W, Michalski JC (2007). Analysis of protein glycosylation by mass spectrometry. Nat. Protoc..

[14] Mechref Y, Novotny MV (2002). Structural investigations of glycoconjugates at high sensitivity. Chem. Rev..

[15] An HJ, Lebrilla CB (2011). Structure elucidation of native N- and O-linked glycans by tandem mass spectrometry (tutorial). Mass Spectrom. Rev..

[16] Ruhaak LR, Zauner G, Huhn C, Bruggink C, Deelder AM, Wuhrer M (2010). Glycan labeling strategies and their use in identification and quantification. Anal. Bioanal. Chem..

[17] Huang Y, Shi X, Yu X, Leymarie N, Staples GO, Yin H, Killeen K, Zaia J (2011). Improved liquid chromatography-MS/MS of heparan sulfate oligosaccharides via chip-based pulsed makeup flow. Anal. Chem..

[18] Geyer H, Geyer R (2006). Strategies for analysis of glycoprotein glycosylation. Biochim. Biophys. Acta.

[19] Domann PJ, Pardos-Pardos AC, Fernandes DL, Spencer DI, Radcliffe CM, Royle L, Dwek RA, Rudd PM (2007). Separation-based glycoprofiling approaches using fluorescent labels. Proteomics.

[20] Ruhaak, L.R., Hennig, R., Huhn, C., Borowiak, M., Dolhain, R.J., Deelder, A.M., Rapp, E., Wuhrer, M.: Optimized workflow for preparation of APTS-labeled N-glycans allowing high-throughput analysis of human plasma glycomes using 48-channel multiplexed CGE-LIF. J. Proteome Res. (2010) 10.1021/pr100802f 20886907

[21] Vanderschaeghe D, Szekrenyes A, Wenz C, Gassmann M, Naik N, Bynum M, Yin H, Delanghe J, Guttman A, Callewaert N (2010). High-throughput profiling of the serum N-glycome on capillary electrophoresis microfluidics systems: toward clinical implementation of GlycoHepatoTest. Anal. Chem..

[22] Marino K, Bones J, Kattla JJ, Rudd PM (2010). A systematic approach to protein glycosylation analysis: a path through the maze. Nat. Chem. Biol..

[23] Royle L, Campbell MP, Radcliffe CM, White DM, Harvey DJ, Abrahams JL, Kim YG, Henry GW, Shadick NA, Weinblatt ME, Lee DM, Rudd PM, Dwek RA (2008). HPLC-based analysis of serum N-glycans on a 96-well plate platform with dedicated database software. Anal. Biochem..

[24] Ahn J, Bones J, Yu YQ, Rudd PM, Gilar M (2010). Separation of 2-aminobenzamide labeled glycans using hydrophilic interaction chromatography columns packed with 1.7 microm sorbent. J. Chromatogr. B Analyt. Technol. Biomed. Life Sci..

[25] Prien JM, Ashline DJ, Lapadula AJ, Zhang H, Reinhold VN (2009). The high mannose glycans from bovine ribonuclease B isomer characterization by ion trap MS. J. Am. Soc. Mass Spectrom..

[26] Stumpo KA, Reinhold VN (2010). The N-glycome of human plasma. J. Proteome Res..

[27] Karlsson NG, Wilson NL, Wirth HJ, Dawes P, Joshi H, Packer NH (2004). Negative ion graphitised carbon nano-liquid chromatography/mass spectrometry increases sensitivity for glycoprotein oligosaccharide analysis. Rapid Commun. Mass Spectrom..

[28] Karlsson NG, Schulz BL, Packer NH (2004). Structural determination of neutral O-linked oligosaccharide alditols by negative ion LC-electrospray-MSn. J. Am. Soc. Mass Spectrom..

[29] Pabst M, Bondili JS, Stadlmann J, Mach L, Altmann F (2007). Mass + retention time = structure: a strategy for the analysis of N-glycans by carbon LC-ESI-MS and its application to fibrin N-glycans. Anal. Chem..

[30] Wuhrer M, de Boer AR, Deelder AM (2009). Structural Glycomics using Hydrophilic Interaction Chromatography (HILIC) with Mass Spectrometry. Mass Spectrom. Rev..

[31] Zauner G, Deelder AM, Wuhrer M (2011). Recent advances in hydrophilic interaction liquid chromatography (HILIC) for structural glycomics. Electrophoresis.

[32] Bruggink C, Wuhrer M, Koeleman CA, Barreto V, Liu Y, Pohl C, Ingendoh A, Hokke CH, Deelder AM (2005). Oligosaccharide analysis by capillary-scale high-pH anion-exchange chromatography with on-line ion-trap mass spectrometry. J. Chromatogr. B.

[33] Harvey DJ (2011). Derivatization of carbohydrates for analysis by chromatography; electrophoresis and mass spectrometry. J. Chromatogr. B Analyt. Technol. Biomed. Life Sci..

[34] Qian J, Liu T, Yang L, Daus A, Crowley R, Zhou Q (2007). Structural characterization of N-linked oligosaccharides on monoclonal antibody cetuximab by the combination of orthogonal matrix-assisted laser desorption/ionization hybrid quadrupole-quadrupole time-of-flight tandem mass spectrometry and sequential enzymatic digestion. Anal. Biochem..

[35] Wuhrer M, Deelder AM (2005). Negative-mode MALDI-TOF/TOF-MS of oligosaccharides labeled with 2-aminobenzamide. Anal. Chem..

[36] Harvey DJ (2005). Fragmentation of negative ions from carbohydrates: part 3. Fragmentation of hybrid and complex N-linked glycans. J. Am. Soc. Mass Spectrom..

[37] Harvey DJ, Jaeken J, Butler M, Armitage AJ, Rudd PM, Dwek RA (2010). Fragmentation of negative ions from N-linked carbohydrates, part 4. Fragmentation of complex glycans lacking substitution on the 6-antenna. J. Mass Spectrom..

[38] Mechref Y, Novotny MV, Krishnan C (2003). Structural characterization of oligosaccharides using MALDI-TOF/TOF tandem mass spectrometry. Anal. Chem..

[39] Guillard M, Gloerich J, Wessels HJ, Morava E, Wevers RA, Lefeber DJ (2009). Automated measurement of permethylated serum N-glycans by MALDI-linear ion trap mass spectrometry. Carbohydr. Res..

[40] Halim, A., Nilsson, J., Ruetschi, U., Hesse, C., Larson, G.: Human urinary glycoproteomics; attachment site specific analysis of N-and O-linked glycosylations by CID and ECD. Mol. Cell. Proteomics (2011) 10.1074/mcp.M111.013649 PMC3322569 22171320

[41] Halim A, Brinkmalm G, Ruetschi U, Westman-Brinkmalm A, Portelius E, Zetterberg H, Blennow K, Larson G, Nilsson J (2011). Site-specific characterization of threonine, serine, and tyrosine glycosylations of amyloid precursor protein/amyloid beta-peptides in human cerebrospinal fluid. Proc. Natl. Acad. Sci. U. S. A..

[42] Nilsson J, Ruetschi U, Halim A, Hesse C, Carlsohn E, Brinkmalm G, Larson G (2009). Enrichment of glycopeptides for glycan structure and attachment site identification. Nat. Methods.

[43] Steentoft C, Vakhrushev SY, Vester-Christensen MB, Schjoldager KT, Kong Y, Bennett EP, Mandel U, Wandall H, Levery SB, Clausen H (2011). Mining the O-glycoproteome using zinc-finger nuclease-glycoengineered SimpleCell lines. Nat. Methods.

[44] Deshpande N, Jensen PH, Packer NH, Kolarich D (2010). GlycoSpectrumScan: fishing glycopeptides from MS spectra of protease digests of human colostrum sIgA. J. Proteome Res..

[45] Christiansen MN, Kolarich D, Nevalainen H, Packer NH, Jensen PH (2010). Challenges of determining O-glycopeptide heterogeneity: a fungal glucanase model system. Anal. Chem..

[46] Zielinska DF, Gnad F, Wisniewski JR, Mann M (2010). Precision mapping of an in vivo N-glycoproteome reveals rigid topological and sequence constraints. Cell.

[47] Mysling S, Palmisano G, Hojrup P, Thaysen-Andersen M (2010). Utilizing ion-pairing hydrophilic interaction chromatography solid phase extraction for efficient glycopeptide enrichment in glycoproteomics. Anal. Chem..

[48] Selman MH, Hemayatkar M, Deelder AM, Wuhrer M (2011). Cotton HILIC SPE microtips for microscale purification and enrichment of glycans and glycopeptides. Anal. Chem..

[49] Wuhrer M, Koeleman CA, Hokke CH, Deelder AM (2006). Mass spectrometry of proton adducts of fucosylated N-glycans: fucose transfer between antennae gives rise to misleading fragments. Rapid Commun. Mass Spectrom..

[50] Wuhrer M, Deelder AM, van der Burgt YE (2011). Mass spectrometric glycan rearrangements. Mass Spectrom. Rev..

[51] Reid CQ, Tait A, Baldascini H, Mohindra A, Racher A, Bilsborough S, Smales CM, Hoare M (2010). Rapid whole monoclonal antibody analysis by mass spectrometry: an ultra scale-down study of the effect of harvesting by centrifugation on the post-translational modification profile. Biotechnol. Bioeng..

[52] Huhn C, Selman MH, Ruhaak LR, Deelder AM, Wuhrer M (2009). IgG glycosylation analysis. Proteomics.

[53] Schirm M, Schoenhofen IC, Logan SM, Waldron KC, Thibault P (2005). Identification of unusual bacterial glycosylation by tandem mass spectrometry analyses of intact proteins. Anal. Chem..

[54] Tsybin YO, Fornelli L, Stoermer C, Luebeck M, Parra J, Nallet S, Wurm FM, Hartmer R (2011). Structural analysis of intact monoclonal antibodies by electron transfer dissociation mass spectrometry. Anal. Chem..

[55] van de Geijn FE, Wuhrer M, Selman MH, Willemsen SP, de Man YA, Deelder AM, Hazes JM, Dolhain RJ (2009). Immunoglobulin G galactosylation and sialylation are associated with pregnancy-induced improvement of rheumatoid arthritis and the postpartum flare: results from a large prospective cohort study. Arthritis Res. Ther..

[56] Ruhaak LR, Uh HW, Beekman M, Koeleman CA, Hokke CH, Westendorp RG, Wuhrer M, Houwing-Duistermaat JJ, Slagboom PE, Deelder AM (2010). Decreased levels of bisecting GlcNAc glycoforms of IgG are associated with human longevity. PLoS One.

[57] Selman, M.H., McDonnell, L.A., Palmblad, M., Ruhaak, L.R., Deelder, A.M., Wuhrer, M.: Immunoglobulin G glycopeptide profiling by matrix-assisted laser desorption ionization fourier transform ion cyclotron resonance mass spectrometry. Anal. Chem. (2010) 10.1021/ac9024413 20058878

[58] Selman, M.H., Derks, R.J., Bondt, A., Palmblad, M., Schoenmaker, B., Koeleman, C.A., van de Geijn, F.E., Dolhain, R.J., Deelder, A.M., Wuhrer, M.: Fc specific IgG glycosylation profiling by robust nano-reverse phase HPLC-MS using a sheath-flow ESI sprayer interface. J. Proteomics. (2011) 10.1016/j.jprot.2011.11.003 22120122

[59] Flangea C, Serb A, Sisu E, Zamfir AD (2011). Reprint of: chip-based nanoelectrospray mass spectrometry of brain gangliosides. Biochim. Biophys. Acta.

[60] Muthing J, Distler U (2010). Advances on the compositional analysis of glycosphingolipids combining thin-layer chromatography with mass spectrometry. Mass Spectrom. Rev..

[61] Levery SB (2005). Glycosphingolipid structural analysis and glycosphingolipidomics. Methods Enzymol..

[62] Bindila L, Peter-Katalinic J (2009). Chip-mass spectrometry for glycomic studies. Mass Spectrom. Rev..

[63] Meisen I, Mormann M, Muthing J (2011). Thin-layer chromatography, overlay technique and mass spectrometry: a versatile triad advancing glycosphingolipidomics. Biochim. Biophys. Acta.

[64] Kirsch S, Muthing J, Peter-Katalinic J, Bindila L (2009). On-line nano-HPLC/ESI QTOF MS monitoring of alpha2-3 and alpha2-6 sialylation in granulocyte glycosphingolipidome. Biol. Chem..

[65] Ohtsubo K, Marth JD (2006). Glycosylation in cellular mechanisms of health and disease. Cell.

[66] An HJ, Kronewitter SR, de Leoz MLA, Lebrilla CB (2009). Glycomics and disease markers. Curr. Opin. Chem. Biol..

[67] Adamczyk, B., Tharmalingam, T., Rudd, P.M.: Glycans as cancer biomarkers. Biochim. Biophys. Acta. (2011) 10.1016/j.bbagen.2011.12.001 22178561

[68] Guillard M, Morava E, van Delft FL, Hague R, Korner C, Adamowicz M, Wevers RA, Lefeber DJ (2011). Plasma N-glycan profiling by mass spectrometry for congenital disorders of glycosylation type II. Clin. Chem..

[69] Guillard M, Morava E, de Ruijter J, Roscioli T, Penzien J, van den Heuvel L, Willemsen MA, de Brouwer A, Bodamer OA, Wevers RA, Lefeber DJ (2011). B4GALT1-congenital disorders of glycosylation presents as a non-neurologic glycosylation disorder with hepatointestinal involvement. J. Pediatr..

[70] Froesch M, Bindila L, Zamfir A, Peter-Katalinic J (2003). Sialylation analysis of O-glycosylated sialylated peptides from urine of patients suffering from Schindler’s disease by Fourier transform ion cyclotron resonance mass spectrometry and sustained off-resonance irradiation collision-induced dissociation. Rapid Commun. Mass Spectrom..

[71] Kruger R, Bruns K, Grunhage S, Rossmann H, Reinke J, Beck M, Lackner KJ (2010). Determination of globotriaosylceramide in plasma and urine by mass spectrometry. Clin. Chem. Lab. Med..

[72] Kruger, R., Tholey, A., Jakoby, T., Vogelsberger, R., Monnikes, R., Rossmann, H., Beck, M., Lackner, K.J.: Quantification of the Fabry marker lysoGb3 in human plasma by tandem mass spectrometry. J. Chromatogr. B Analyt. Technol. Biomed. Life Sci. (2011) 10.1016/j.jchromb.2011.11.020 22138589

[73] Bruggink C, Poorthuis BJ, Piraud M, Froissart R, Deelder AM, Wuhrer M (2010). Glycan profiling of urine, amniotic fluid and ascitic fluid from galactosialidosis patients reveals novel oligosaccharides with reducing end hexose and aldohexonic acid residues. FEBS J..

[74] Domon B, Costello C (1988). A systematic nomenclature for carbohydrate fragmentation in FAB-MS/MS spectra of glycoconjugates. Glycoconj. J..

[75] Balog, C.I., Mayboroda, O.M., Wuhrer, M., Hokke, C.H., Deelder, A.M., Hensbergen, P.J.: Mass spectrometric identification of aberrantly glycosylated human apolipoprotein C-III peptides in urine from Schistosoma mansoni-infected individuals. Mol. Cell. Proteomics (2010) 10.1074/mcp.M900537-MCP200 PMC2860228 20071361

[76] Pacchiarotta T, Hensbergen PJ, Wuhrer M, van Niewkoop C, Nevedomskaya E, Derks RJ, Schoenmaker B, Koeleman CA, van Dissel J, Deelder AM, Mayboroda OA (2012). Fibrinogen alpha chain O-glycopeptides as possible markers of urinary tract infection. J. Proteomics.

[77] Kyselova Z, Mechref Y, Al Bataineh MM, Dobrolecki LE, Hickey RJ, Vinson J, Sweeney CJ, Novotny MV (2007). Alterations in the serum glycome due to metastatic prostate cancer. J. Proteome Res..

[78] Kyselova Z, Mechref Y, Kang P, Goetz JA, Dobrolecki LE, Sledge GW, Schnaper L, Hickey RJ, Malkas LH, Novotny MV (2008). Breast cancer diagnosis and prognosis through quantitative measurements of serum glycan profiles. Clin. Chem..

[79] Imre T, Kremmer T, Heberger K, Molnar-Szollosi E, Ludanyi K, Pocsfalvi G, Malorni A, Drahos L, Vekey K (2008). Mass spectrometric and linear discriminant analysis of N-glycans of human serum alpha-1-acid glycoprotein in cancer patients and healthy individuals. J. Proteomics.

[80] Gornik, O., Pavic, T., Lauc, G.: Alternative glycosylation modulates function of IgG and other proteins - implications on evolution and disease. Biochim. Biophys. Acta. (2011) 10.1016/j.bbagen.2011.12.004 22183029

[81] Wang J, Balog CI, Stavenhagen K, Koeleman CA, Scherer HU, Selman MH, Deelder AM, Huizinga TW, Toes RE, Wuhrer M (2011). Fc-glycosylation of IgG1 is modulated by B-cell stimuli. Mol. Cell. Proteomics.

[82] Wuhrer M, Porcelijn L, Kapur R, Koeleman CA, Deelder AM, de Haas M, Vidarsson G (2009). Regulated glycosylation patterns of IgG during alloimmune responses against human platelet antigens. J. Proteome Res..

[83] Iida S, Misaka H, Inoue M, Shibata M, Nakano R, Yamane-Ohnuki N, Wakitani M, Yano K, Shitara K, Satoh M (2006). Nonfucosylated therapeutic IgG1 antibody can evade the inhibitory effect of serum immunoglobulin G on antibody-dependent cellular cytotoxicity through its high binding to FcgammaRIIIa. Clin. Cancer Res..

[84] Anthony RM, Kobayashi T, Wermeling F, Ravetch JV (2011). Intravenous gammaglobulin suppresses inflammation through a novel T(H)2 pathway. Nature.

[85] Tran JC, Zamdborg L, Ahlf DR, Lee JE, Catherman AD, Durbin KR, Tipton JD, Vellaichamy A, Kellie JF, Li M, Wu C, Sweet SM, Early BP, Siuti N, LeDuc RD, Compton PD, Thomas PM, Kelleher NL (2011). Mapping intact protein isoforms in discovery mode using top-down proteomics. Nature.

[86] Jungblut PR, Holzhutter HG, Apweiler R, Schluter H (2008). The speciation of the proteome. Chem. Cent. J..

